# Semantics impacts response to phonics through spelling intervention in children with dyslexia

**DOI:** 10.1007/s11881-021-00233-1

**Published:** 2021-06-22

**Authors:** Robin van Rijthoven, Tijs Kleemans, Eliane Segers, Ludo Verhoeven

**Affiliations:** 1 Behavioural Science Institute, Radboud University, Nijmegen, The Netherlands; 2 OPM, Nijmegen, The Netherlands

**Keywords:** Dyslexia, Phonics through spelling intervention, Response to intervention, Semantics, Spelling errors

## Abstract

We examined the response to a phonics through spelling intervention in 52 children with dyslexia by analyzing their phonological, morphological, and orthographical spelling errors both before and after the intervention whereas their spelling errors before the intervention were compared with those of 105 typically developing spellers. A possible compensatory role of semantics on the intervention effects was also investigated. Results showed that before the intervention, children with dyslexia and the typically developing children both made most morphological errors, followed by orthographic and phonological errors. Within each category, children with dyslexia made more errors than the typically developing children, with differences being largest for phonological errors. Children with dyslexia with better developed semantic representations turned out to make less phonological, morphological, and orthographic errors compared with children with dyslexia with less developed semantic representations. The intervention for children with dyslexia led to a reduction of all error types, mostly of the orthographic errors. In addition, semantics was related to the decline in phonological, morphological, and orthographic spelling errors. This study implicates that semantic stimulation could benefit the spelling development of children at risk for or with dyslexia.

The spelling of phonologically transparent words is rather straightforward. Starting from the phonemic segmentation of a word, the speller applies phoneme-grapheme correspondence rules to arrive at the correct spelling (Vanderswalmen et al., 2010). For words that are phonologically less transparent, the speller may use a strategy to search for analogy with similar spellings of known words, building on previously acquired phonological and orthographic representations (Allen, 2019). Research shows that children with dyslexia have severe problems in building up these representations (Lyon et al., 2003), which can be explained by their phonological deficit (Conrad, 2008; Göbel & Snowling, 2010). Consequently, children with dyslexia make more phonological errors in reading as well as in spelling compared to typically developing children (e.g., Bourassa et al., 2006). In addition, they also make more orthographic and morphological spelling errors. This can be explained from the fact that orthographic and morphological spelling builds upon the phonological base (Nunes et al., 1997). An overall delay in spelling development in children with dyslexia showing a phonological deficit that persists into adolescence can thus be expected (see, e.g., Bourassa & Treiman, 2003). Given that variation in semantic abilities has previously been shown to impact reading (Nation & Snowling, 2004; Torppa et al., 2010; van Bergen et al., 2014) and spelling ability (Tainturier & Rapp, 2001; Ouellette, 2010), it might well be the case that children with dyslexia may compensate their low spelling outcomes through semantics. However, semantics has not yet been included as a predictor in responsiveness to intervention studies. Therefore, in the present study, the impact of semantics on a response to phonics through spelling intervention in children with dyslexia was examined.

## Comparison of learning to spell for children with and without dyslexia

In alphabetic writing systems, learning to spell starts with the segmentation of spoken words into singular sounds, followed by phoneme-to-grapheme mapping and word synthesis (Vanderswalmen et al., 2010). This so-called phonetic strategy helps to build up bidirectional connections between phonological and orthographic representations (Bosman & van Orden, 1997). Strong and bidirectional connections could facilitate a self-teaching mechanism (Share, 1995) to foster spelling development (Burt & Tate, 2002).

Learning to spell, however, requires more than one-to-one phoneme-grapheme mapping. After mastering this phonetic strategy, children encounter words that are orthographically more complex. In this phase, word spelling inconsistencies need to be learned in order to become a skilled speller (Ehri, 2000; Treiman, 2018). Specific knowledge of morphological and orthographic patterns is then connected to already existing semantic representations, so that analogies of common words can be used in the spelling of uncommon words (Allen, 2019; Treiman et al., 1994). The accumulating knowledge of morphological and orthographic patterns further strengthens the quality of lexical representations. Indeed, it has been found that high-quality phonological, orthographic, and semantic representations lead to better spelling levels (Perfetti, 2007). Building up high-quality phonological and orthographic representations is demanding for all beginning spellers (as described by Cassar et al., 2005). However, for children with dyslexia, learning to spell words correctly is even more challenging (Lyon et al., 2003), due to a phonological deficit (Conrad, 2008; Göbel & Snowling, 2010). This deficit makes it difficult to learn and retrieve phonological information from long-term memory (Cassar et al., 2005), resulting in more phonological, morphological, and orthographic spelling errors as compared to typically developing children (Bourassa & Treiman, 2003; Bourassa et al., 2006; Cassar et al., 2005).

The phonological deficit is an important predictive factor for spelling development (Wimmer & Mayringer, 2002). However, as proposed by the phonological-core variable differences model by Stanovich (1988), along with the core phonological deficit, other variables influence the spelling development as well. This model posits that all poor readers have a phonological deficit, but that additional cognitive factors are important for their reading and spelling skills. The triangular framework (Seidenberg & McClelland, 1989) highlights the importance of high-quality semantic representations for learning to read and spell. In line with this model, it can be assumed that variation in semantic abilities could partly compensate for the phonological deficit, especially since the semantic abilities of children with dyslexia are quite similar to those of typically developing children (Nation & Snowling, 1998). Phonological and semantic skills influence reading development from its earliest stages and influence each other as well (e.g., Laing & Hulme, 1999). However, Nation and Snowling (2004) showed that within the typically developing readers, broader language skills (also verbal production and comprehension) beyond phonology make important contributions to word recognition development. Prior research regarding reading indeed showed the benefits of well-developed semantic representations (e.g., Nation & Snowling, 2004; Torppa et al., 2010) and suggested a possible importance of semantic representations for spelling as well (Ouellette, 2010; Ouellette & Fraser, 2009). It remains, however, unclear to what extent variation in semantic ability impacts phonological versus orthographic spelling of children with dyslexia on top of phonological abilities.

## Analysis of spelling errors

Only few studies addressed the distribution of different types of spelling errors (e.g., Bourassa & Treiman, 2003) and none predicted spelling errors from individual differences in cognitive factors. One way of addressing phonological and orthographic errors separately is to analyze and categorize spelling errors. Spelling error analyses and the comparisons between typical spellers and spellers with dyslexia are mostly based on post hoc error classifications. The errors are subdivided into three broad categories in order to define the source of the errors: phonological, morphological, and orthographic errors (see Moats, 1995; Sawyer et al., 1999; Tops et al., 2014; Vanderswalmen et al., 2010). Morphological and orthographic errors represent the morphological and orthographic patterns that are part of the orthographic spelling development (Allen, 2019). In phonological errors, a difference occurs between the pronunciation of the written word and the spoken target word. In morphological errors, words maintain the proper pronunciation but are misspelled based on language-specific grammatical rules. In orthographic errors, words also maintain the proper pronunciation but are misspelled based on orthographical conventions or based on the original language a word is from (see Appendix Table 6).

Previous research that categorized spelling errors into these categories concluded that children with dyslexia proportionally made the same mistakes, but in higher numbers compared to typically developing children (Bourassa & Treiman, 2003; Bourassa et al., 2006). As a case in point, Bourassa and Treiman (2003) found that both typically developing children and children with dyslexia made mostly linguistically motivated errors. Errors made by children with dyslexia with an average age of 11 years were comparable with mistakes of typically developing children that were on average 3.5 years younger. This is, however, not just a temporarily delay or slow development, but a delay that persists in later life. Indeed, Tops et al. (2014) showed that students with dyslexia in higher education, despite their ongoing effort, still made more spelling errors than their peers without dyslexia. However, relative differences between all three error types were not studied yet in children, since Bourassa and Treiman (2003) and Bourassa et al. (2006) did not include all three error types.

## Interventions for spelling development

The weak spelling performances of children with dyslexia compared to typically developing children have serious consequences for their general development at school. For instance, when one is still struggling with spelling correctly, working memory cannot be fully used to write down a story in a developed manner (Wakely et al., 2006). This influences text production fluency and quality (Berninger & Swanson, 1994; McCutchen, 2000). Early intervention is needed in order to give these children the same opportunities in life. These interventions are a good way of studying the spelling development of children with dyslexia.

In such interventions, it is recommended to include and explicitly teach phonological, morphological, and orthographic awareness of word forms, their parts, and their interrelationships (Berninger et al., 2008; Galuschka et al., 2020). As concluded by Cassar et al. (2005), children with dyslexia need direct assistance to develop their spelling skills by improving both the phonological and orthographic representations. Skilled spelling requires a solid base of grapheme-phoneme translation which enables the formation of strong bidirectional relations between phonological and orthographic representations. Next, a combination of instruction and experience in both reading and spelling can help to learn the increasingly complex and specific orthographic spelling by learning morphological and orthographic patterns (Caravolas et al., 2001). This further enhances the quality of the relation between phonological and orthographic representations and includes semantic features as well (Perfetti & Hart, 2002).

A specific form of intervention that combines the above described principles is the phonics through spelling intervention. Phonics interventions are in general effective in enhancing spelling (and reading) development in typically developing children, as well as for children with dyslexia (Galuschka et al., 2014). A phonics through spelling intervention includes more time for instruction and practice in spelling compared to other phonics interventions. By doing so, the intervention enhances both reading and spelling levels, probably because of the high-quality network of phonological, orthographical, and semantic representations that are formed during the intervention (Van Rijthoven et al., 2020). However, results of phonics interventions on spelling have only been expressed in the amount of words written correctly, whereas comparing spelling error profiles before and after the intervention could give new insights in the phonological and orthographic spelling development due to the intervention.

Despite the fact that children with dyslexia have shared problems, large individual differences were found in the progress during intervention (e.g., Galuschka et al., 2014). Individual differences during intervention were mainly caused by a phonological deficit (see Snowling, 1998; Tilanus et al., 2019). However, no distinction was made between phonological and orthographic spelling and semantics was not included as a predictor.

## The present study

Previous research showed that children with dyslexia make more spelling errors compared to typically developing children. In order to enhance the possibilities of children with dyslexia early in life, it is important to gain more insight in the relative differences between spelling error patterns these children show both before and after an intervention and in the extent to which the number and distribution of errors is related to children’s semantic abilities. In the present study, the following research questions were asked:

(a) To what extent do children with dyslexia differ from typically developing children in phonological and orthographical spelling?
  (b) What is the role of semantics in predicting the phonological and orthographical spelling of children with dyslexia?
(a) To what extent can phonological and orthographical spelling abilities of children with dyslexia be trained through a phonics through spelling intervention?
  (b) What is the role of semantics in predicting the development of phonological and orthographical spelling in children with dyslexia due to a phonics through spelling intervention?

Regarding the first research question, firstly, we expected children with dyslexia to make more but not proportionally different errors. Second, we expected orthographic spelling to be more difficult than phonological spelling for both children with dyslexia and typically developing children. Third, we expected semantics to be a compensatory factor for children with dyslexia across error types.

Regarding the second research question, we expected a decline in the amount of errors in all categories with no relative differences between the proportion of errors due to the intervention. Furthermore, we expected a decline in the amount of errors across the three types to be predicted by the children’s level of semantic abilities.

## Method

### Participants

Participants were children diagnosed with developmental dyslexia (34 boys, 18 girls) and typically developing children (52 boys, 53 girls*).* All participants spoke Dutch as their primary language and all parents gave active consent to use the collected data for research purposes.

The children with dyslexia were diagnosed between 2009 and 2013 following the protocol by Blomert (2006), which is in line with the definition of dyslexia of the International Dyslexia Association (2002). The Dutch protocol for diagnosis of developmental dyslexia (Blomert, 2006) states that teachers have to prove persistent reading problems (resistance to treatment) and severity (weak performances on word reading and spelling during one and a half school year). In the subsequent diagnosis, a phonological deficit needs to be evidenced and other explanations of reading or spelling problems excluded by a certified clinical psychologist. After formally being diagnosed with dyslexia, children received an in-service phonic through spelling intervention in a clinic for assessment and intervention for children with learning difficulties. The mean age of this group of children at the start of the assessment was 8.97 years (*SD* = .94). Children were in grade 2 (*n* = 17), grade 3 (*n* = 23), grade 4 (*n* = 9), grade 5 (*n* = 2), and grade 6 (*n* = 1). All children had semantics within the normal range both in total scores (mean = 108.35, *SD* = 12.18) and standardized subtest scores (m_information_ = 10.50, *SD*_information_ = 2.42, m_simularities_ = 12.25, *SD*_simularities_ = 2.83, m_vocabulary_ = 11.31, *SD*_vocabulary_ = 2.32, m_comprehension_ = 11.58, *SD*_comprehension_ = 2.41).

The mean age of the children in the control group was 8.88 years (*SD =* .89). Children were in grade 2 (*n* = 25), grade 3 (*n* = 52), and grade 4 (*n* = 28). All children had semantics within the normal range both in total scores (mean = 101.67, *SD* = 13.84) and standardized subtest scores (m_information_ = 10.40, *SD*_information_ = 2.63, m_simularities_ = 10.98, *SD*_simularities_ = 2.81, m_vocabulary_ = 10.46, *SD*_vocabulary_ = 2.82, m_comprehension_ = 9.34, *SD*_comprehension_ = 2.81). The fact that both groups scored within the normal range on the four measures of the semantic ability seems to converge with the aptitude-achievement discrepancy model that defined dyslexia as a discrepancy between rather normal intelligence and weak reading scores (Fletcher, Lyon, Fuchs, & Barnes, 2007).

### Procedure

With respect to the recruitment of the control group, five schools for mainstream primary education throughout the Netherlands were asked by letter and telephone to participate in the present study. When a school agreed to participate, parents gave active consent to let their child participate in the present study.

With respect to the group of children with dyslexia, the data from the current study was based on existing data collected by a clinic for assessment and intervention of children with learning disorders. All children were tested between 2009 and 2013 in two consecutive mornings by clinicians on rapid automatized naming, phonological awareness, verbal working memory, pseudoword decoding, word decoding, spelling, and semantics. Two or 3 weeks after the assessment, the phonics through spelling intervention started. After the intervention, all participants received the posttest, including pseudoword decoding, word decoding, and spelling measures. All children from the control group were tested once, during one school day.

### Outcome measures

#### Spelling

Spelling was measured with the standardized “PI word dictation” (Geelhoed & Reitsma, 1999). In this task, children were asked to write single Dutch words correctly. The dictation consisted of 135 words, divided into 9 blocks of 15 words. First, a sentence was read aloud and afterwards, the target word was repeated. The test was terminated when a child failed to write at least eight words correctly within one block. The number of correctly written words was counted. There were two versions available of the test (version A and version B). The reliability of both version A and version B differs per age but is at least .90 (Geelhoed & Reitsma, 1999).

### Predictor measures

#### Phonological awareness

Two subtests from the “Screening Test for Dyslexia” (Kort et al., 2005a) were used. First, during “Phoneme Deletion,” children were asked to omit a phoneme from an orally presented word and speak out the remaining word (e.g., “da*k*” [roof] minus “*k*” [f] is “da” [roo]). Testing was terminated after four consecutive mistakes. Second, during the subtest “*Spoonerism*,” children had to switch the first sounds of two words (e.g., “*J*ohn *L*ennon” becomes “*L*ohn *J*ennon”). Testing was terminated after five consecutive mistakes. The reliability differs per age but is at least .60.

#### Rapid automatized naming

RAN was measured using two subtests of “Continuous Naming and Reading Words” (van den Bos & Lutje Spelberg, 2010). During “*Naming Letters*,” children had to read out loud 50 letters. During “*Naming Digits*,” they were asked to read out loud 50 digits. Children were asked to name these visual stimuli as quickly as possible. The time in seconds needed to finish each subtest was used for analysis which means that a higher score reflects a weaker performance on RAN. The reliability of this measure differs per age but is at least .75.

#### Verbal working memory

Verbal working memory was measured using the backward task of the Number Recall subtest from the Wechsler Intelligence Scale for Children-III (WISC-III^NL^) (Kort et al., 2005b). In this task, the experimenter pronounces sequences of digits that the child was asked to repeat in backward order. Testing was terminated after two consecutive mistakes. The number of correctly recalled sequences was counted. The reliability of this measure differs per age but is at least .50.

#### Semantics

Semantics were measured by adding the z-scores of four subtests from the WISC-III^NL^ (Kort et al., 2005b). Based on the manual, the child received zero, one, or two points for each item. Testing was terminated after four or five (*Information*) consecutive mistakes. The reliability differs per age but is between .64 and .77 (Kort et al., 2005b). First, during “*Information*,” the child has to answer verbally asked questions to test their general knowledge about events, objects, places, and people. Secondly, during “*Similarities*,” the child has to name the similarity between two concepts. Thirdly, during “*Productive vocabulary*,” the experimenter pronounces a word and the task of the child was to define the given word. Fourthly, during “*Comprehension*,” the experimenter asked questions about social situations or common concepts. Kaufman (1975) already showed that these four measures together form a factor named “verbal comprehension,” which is also the case in the current sample (van Rijthoven et al., 2018).

### Spelling error classification

The PI word dictation consisted of 135 words. However, for most children, testing was terminated earlier. We therefore selected the first four blocks (60 words). All possible types of errors within these 60 words were listed and labeled (e.g., phoneme addition, end d/t, ei/ij). These errors were divided into three categories: phonological, morphological, and orthographic errors following Tops et al. (2014), Vanderswalmen et al. (2010), and Worthy and Viise (1996), see Appendix Table 6. Some types of errors could not be classified exclusively into phonological, morphological, or orthographic errors. Words containing these types of errors were removed from the dataset, see Appendix Table 7 (i.e., 21.66% (version A) and 18.33% (version B)). After the removal of the above-mentioned words, the total amount of possible errors was calculated based on the descriptions of the total amount of possible errors within each category. This was done for each version of the PI word dictation (versions A and B) to correct for any differences between the two versions.

Next, the dictation tasks of all participants were screened on the amount and type of errors made by the child. Following Tops et al. (2014), the error classification was based on the end-product and not on the strategy used by the child. For each child with dyslexia, two dictations were screened (pre- and posttest). For typically developing children, a single dictation was screened (all version A). The inter-rater reliability of the two MSc students who did the screening was good: Cohen’s kappa is .84 (A-version) and .88 (B-version).

For each dictation task, all errors were entered in a dataset in which each error was assigned to the type of error. One word could contain multiple errors following the descriptions in Appendix Table 6. In case of early termination of the task (after eight errors in a block of 15 words), all possible errors in non-written words were entered in the dataset based on the assumption that the upcoming words would be too difficult for the child. This is following procedures of other tests such as the WISC-III^NL^ (Kort et al., 2005a) or the PPVT (Dunn & Dunn, 1997). In the end, the total amount of phonological, morphological, and orthographic errors was calculated by adding the types of errors as these have been classified in Appendix Table 6. Finally, the percentage of errors for each classification was calculated based on the total amount of possible errors for each category per version.

### Phonics through spelling intervention

A phonics through spelling intervention aims to reach a functional level of technical reading and spelling by means of combining reading and writing in one intervention following the protocol by Blomert (2006). Unique to a phonics through spelling intervention is that during the intervention, both reading and spelling instruction and exercises were equally balanced in terms of spent time (50–50). This is rather unique as most studies include less or even no spelling instruction or exercises. Children had a weekly 45-min session with a clinician. The mean length of the intervention was 27.06 weeks (SD = 4.79). Variation in the length of the intervention occurred due to variation in the post-intervention assessment schedule (for instance due to holidays or personal circumstances). Furthermore, variation in the length of the intervention occurred due to variation in time needed to acquire 80% accuracy levels and improved fluency levels as described below. During the sessions, the clinician tailored the intervention as much as possible to each child’s needs. Explicit direct instruction, guided exercises, and feedback were given according to each child’s needs. Approximately half of the time was spent on reading activities and the other half of the time was spent on spelling activities. The continuity of quality during assessment and intervention was guaranteed by supervision of certified clinical health psychologists. The intervention included two stages:

1. Phonological spelling

The intervention started with practice of the phonological base of reading and spelling due to learning the grapheme-phoneme correspondences (GPCs). After learning the GPCs, children learned to use this letter knowledge in reading and writing words and sentences/texts by using an explicit strategy. When children mastered the basic levels, children learned to read and write words based on syllables as well. Accuracy was trained first, followed by efficiency and words and sentences/texts increased in difficulty. Feedback was given on accuracy and later also on efficiency.

2. Orthographic spelling

Dutch is a rather transparent language, but still morphological rules and orthographic patterns need to be learned to write and read words (mostly polysyllabic words) correctly. The morphological rules and orthographic patterns can be found in Appendix Table 6 and were taught according to each child’s needs.

In order to rehearse the above-mentioned spelling and reading knowledge, children had to do home exercises for reading and spelling. Parents were asked to train four times a week during 15 min with prescribed exercises. All parents have confirmed that this has been complied with. Parents reflected on the home exercises in a day-to-day logbook. When a child reached an accuracy of 80% during practice (read or write 80% of the words correctly) and improved significant in their fluency (more fluent compared to the first time words were read), the clinician moved on to the next topic of intervention. This formative testing was sustained throughout the entire intervention. Therefore, variation in the length of the program is present.

## Results

### Individual differences in phonological and orthographic spelling

Research question 1a was about the differences between children with dyslexia and typically developing children regarding their phonological and orthographical spelling. Table 1 presents the descriptive statistics.

**Table 1:** Descriptive statistics for children with dyslexia at pretest (N = 52) and typically developing children (N = 105)

		Children with dyslexia		Typically developing children	
		*M*	SD	*M*	SD
Outcome measures					
Word spelling pretest	Raw scores	43.15	23.54	80.23	20.95
Phonological errors	Raw scores	103.34	160.77	1.98	2.50
	Percentages	16.30	25.24	0.32	0.41
Morphological errors	Raw scores	5.69	4.93	1.88	2.14
	Percentages	33.74	28.91	11.72	13.36
Orthographical errors	Raw scores	17.19	22.41	1.00	1.61
	Percentages	20.63	26.42	1.27	2.04
Predictor measures					
Phonological awareness					
Spoonerism	Raw scores	2.80	3.35	6.99	3.40
Phoneme deletion	Raw scores	7.96	2.35	9.99	1.74
Rapid automatized naming					
Letter naming	Raw scores	41.63	10.94	31.00	10.37
Digit naming	Raw scores	37.79	11.26	28.21	6.98
Verbal working memory	Raw scores	3.92	1.31	4.59	1.65
Semantics					
Information	Raw scores	12.58	3.27	12.64	3.23
Similarities	Raw scores	14.27	2.42	12.49	3.68
Vocabulary	Raw scores	30.23	6.14	28.22	6.94
Comprehension	Raw scores	20.60	5.52	16.60	5.62

To compare children with dyslexia and typically developing children, we performed *t-*tests for independent samples with Holm-Bonferroni correction (Holm, 1979). Regarding the outcome measures, children with dyslexia scored below typically developing children in the total amount of words written correctly in the dictation task (*t*(155) = −10.011, *p* < .001, *d* = 1.66) and made higher percentages of errors in all three categories (phonological errors *t*(141) = 4.565, *p* < .001, *d* = 0.90; morphological errors *t*(62) = 4.565, *p* < .001, *d* = 0.98; orthographic errors *t*(155) = 5.278, *p* < .001, *d* = 1.03). Regarding the predictive measures, children with dyslexia scored weaker compared to typically developing children on phonological awareness measures (spoonerism *t*(154) = −5.483, *p* < .001, *d* = 1.24; phoneme deletion *t*(154) = −7.249, *p* < .001, *d* = 0.98), rapid automatized naming (letter naming *t*(155) = 5.939, *p* < .001, *d* = 1.00, digit naming *t*(71) = 5.623, *p* < .001, *d* = 1.02), and verbal working memory (*t*(125) = −2.757, *p* = .007, *d* = 0.45). With regard to semantics, the groups scored equally on information (*t*(155) = 0.111, *p* = .911, *d* = 0.02) and vocabulary (*t*(155) = −1.775, *p* = .08 , *d* = 0.31), whereas children with dyslexia outperformed typically developing children on similarities (*t*(155) = 2.611, *p* = .010, *d* = 0.57) and comprehension (*t*(155) = 4.271, *p* < .001, *d* = 0.72).

Next, differences in distribution of errors within each group were studied using ANOVA with Errortype (phonological errors, morphological errors, and orthographical errors) as within subject factor and group (typically developing children, children with dyslexia) as between subject factor. Mauchly’s test indicated that the assumption of sphericity had been violated for the main effects of Errortype, χ^2^ (2) = 0.097, *p <* .001. Therefore, degrees of freedom were corrected using Greenhouse-Geisser estimates of sphericity (*ε* = .53 for Errortype). There was a main effect of Errortype, *F*(1.051, 162.899) = 145.309, *p* < .001, η^2^_p_ = .484, as well as an interaction between Errortype and group, *F*(1, 155) = 5.630, *p* = .017, η^2^_p_ = .035. Children with dyslexia made more phonological (*t*(141) = 4.565, *p* < .001, *d* = 0.90), morphological (*t*(62) = 4.565, *p* < .001, *d* = 0.98), and orthographic errors (*t*(155) = 5.278, *p* < .001, *d* = 1.03) than typically developing children. The interaction suggests that this difference tended to be more pronounced in the phonological errors. Indeed, planned contrasts revealed that the difference between the two groups is larger for phonological and orthographic errors (*F*(1, 155) = 66.45, *p* < .001) and phonological and morphological errors (*F*(1, 155) = 7.27, *p* < .008) compared to morphological and orthographic errors (*F*(1, 155) = 1.54, *p* = .217). Both groups made most morphological errors, followed by orthographic errors and phonological errors.

### Predicting individual differences in phonological and orthographic spelling

Research question 1b was about the role of semantics in predicting the phonological and orthographical spelling of children with dyslexia. To answer this question, hierarchical regression analyses were conducted on all three outcome measures (i.e., phonological errors, morphological errors, and orthographic errors). For the WISC-III measures, given that we had a control group that was matched on age, we chose to use raw scores instead of standardized scores as it would allow us to compare impact of each measure. In step 1, phonological awareness, rapid automatized naming, and verbal working memory were entered, followed by semantics in step 2. Semantics was added separately to see the unique contribution of semantics beyond phonological awareness, rapid automatized naming, and verbal working memory. Correlations between all used measures are presented in Table 2.

**Table 2:** Pearson correlations between the outcome measures (i.e., word spelling, phonological errors, morphological errors, and orthographical errors) and predictor measures (i.e., phonological awareness, rapid automatized naming, verbal working memory, and semantics). Below the diagonal: children with dyslexia (n = 52); above the diagonal: typically developing children (n = 105)

	1.	2.	3.	4.	5.	6.	7.	8.	9.	10.	11.	12.
1. Word spelling raw scores pretest	-	−.585**	−.747**	−.617**	.610**	−.418**	.426**	.563**	-	-	-	-
2. Percentages phonological errors	−.768**	-	.392**	.370**	−.491**	.360**	−.146	−.343**	-	-	-	-
3. Percentages morphological errors	−.870**	.891**	-	.564**	−.519**	.241*	−.227*	−.451**	-	-	-	-
4. Percentages orthographical errors	−.804**	.993**	.891**	-	−.337**	.323**	−.242*	−.283**	-	-	-	-
5. Phonological awareness	.305*	−.245	−.258	−.256	-	−.255**	.317**	.528**	-	-	-	-
6. Rapid automatized naming	−.378**	.239	.301*	.253	−.212	-	−.231*	−.318**	-	-	-	-
7. Verbal working memory	.294*	−.229	−.230	−.234	.088	.040	-	.410**	-	-	-	-
8. Semantics	.652**	−.457**	−.617**	−.482**	.345*	−.359**	.085	-	-	-	-	-
9. Change per session phonological errors	.720**	.893**	−.838**	−.877**	.314*	−.250	.275*	.527**	-	-	-	-
10. Change per session morphological errors	.608**	−.665**	−.811**	−.635**	.117	−.098	−.253	.407**	.771	-	-	-
11. Change per session orthographical errors	.704**	−.879**	−.828**	−.875**	.324*	−.272	−.252	.534**	.991**	.737**	-	-
12. Change per session spelling percentiles	−.255	−.206	−.207	−.217	.292*	−.286*	.023	.357**	.279	.157	.282	-

*p < .05. **p < .01. ***p < .001

The results of the analyses (see Table 3) showed that rapid naming predicted morphological errors (step 1). In step 2, the effect of rapid naming on morphological errors was no longer present and higher scores on semantics resulted in less phonological, morphological, and orthographical errors.

**Table 3:** Cognitive predictors of spelling errors in children with dyslexia (N = 52)

		Phonological errors	Morphological errors			Orthographical errors	
		*ΔR*^*2*^	β	*ΔR*^*2*^	β	*ΔR*^*2*^	β
Step 1		.151		.187*		.163*	
	Phonological awareness		−2.712		−3.089		−2.971
	Rapid automatized naming		2.741		4.065*		3.039
	Verbal working memory		−7.074		−8.265		−7.538
Step 2		.123**		.260***		.137**	
	Phonological awareness		−1.067		−0.359		−1.152
	Rapid automatized naming		1.193		1.495		1.328
	Verbal working memory		−6.300		−6.980		−6.683
	Semantics		−2.811**		−4.666***		−3.107**
Total *R*^2^_adj_		.211**		.399***		.240**	

*p < .05. **p < .01. ***p < .001

To check whether age had any impact on the results, we reran the hierarchical regression analysis including age in step 1 and the predictor measures in steps 2 and 3; results remained similar.

### Change in spelling errors due to intervention in children with dyslexia

Research question 2a focused on the extent to which children with dyslexia develop their phonological and orthographical spelling due to a phonics through spelling intervention.

We first calculated the mean change per session by subtracting the amount of phonological, morphological, and orthographical errors at pre- and posttest to control for the variation in length of the intervention. Following Gollwitzer et al. (2014), change scores are reliable when standard deviations differ between measurement occasions and there needs to be a non-zero variation in observed difference scores. Our data met these requirements. In order to rule out the effects of variation in length of the intervention and to ensure the analyses had enough statistical power, the individual change score was divided by the number of sessions the intervention for each individual lasted. Paired t-tests with Holm-Bonferroni correction (Holm, 1979) were computed to test the differences between pre- and posttest on overall spelling performance and each type of spelling error, respectively. Results showed an increase in overall spelling scores as well as a decrease in all three error types after intervention (see Table 4). Regarding the relative differences in change per session, results showed no differences between phonological and morphological (*t*(45) = 1.604, *p* = .116, *d* = 0.16) and morphological and orthographic errors (*t*(45) = −.614, *p* = .542, *d* = 0.06), whereas the differences of change per session between phonological and orthographic errors were significant (*t*(45) = 4.590, *p* < .001, *d* = 0.10). The change per session is higher for orthographical errors than it is for phonological errors.

**Table 4:** Descriptive statistics from both pre- and posttest on all dependent measures, the results on the t-tests for paired samples, Cohen’s d, and the change per session

	Pretest (n = 52)	Posttest (n = 46)		Difference scores (t-tests)		Change per session		
	*M*	SD	*M*	SD	*t*	*d*	*M*	SD
Word spelling percentiles	5.40	10.50	13.25	15.10	3.41***	0.60	.26	0.56
Percentage of phonological errors	16.30	25.24	1.99	11.07	−4.34***	0.73	−.62	0.98
Percentage of morphological errors	33.74	28.91	14.57	17.06	−5.19***	0.71	−.78	1.05
Percentage of orthographical errors	20.62	26.41	3.78	12.81	−4.83***	0.73	−.72	1.02

*p < .05. **p < .01. ***p < .001

### Predicting individual differences in change scores in children with dyslexia

Finally, research question 2b focused on the predictive role of semantics on the phonological and orthographical spelling development of children with dyslexia due to a phonics through spelling intervention. This question was answered by conducting three hierarchical regression analyses (i.e., one analysis for each change score in error type). For each analysis, the predictor variables (i.e., phonological awareness, rapid automatized naming, and verbal working memory) were entered in step 1, followed by semantics in step 2 to see what the unique contribution of semantics is beyond phonological awareness, rapid automatized naming, and verbal working memory. In step 1, no variables significantly predicted any change score, although the model was significant for phonological and orthographic errors. In step 2, semantics was a positive predictor for all three change scores: the better the semantics, the higher the change scores in phonological, orthographical, and morphological errors (see Table 5).

**Table 5:** Cognitive predictors of the change in spelling errors during the interventions (N = 46)

		Change in phonological errors		Change in morphological errors		Change in orthographical errors	
		*ΔR*^*2*^	β	*ΔR*^*2*^	β	*ΔR*^*2*^	β
Step 1		.212*		.094		.215*	
	Phonological awareness		0.053		0.023		0.057
	Rapid automatized naming		−0.010		−0.004		−0.011
	Verbal working memory		0.200		0.217		0.187
Step 2		.139**		.117*		.139**	
	Phonological awareness		0.024		−0.005		0.027
	Rapid automatized naming		−0.005		< 0.000		−0.007
	Verbal working memory		0.166		0.183		0.151
	Semantics		0.024**		0.24*		0.025**
Total *R*^2^_adj_		.285**		.132*		.290**	

*p < .05. **p < .01. ***p < .001

We again checked whether age had any impact on the results and reran the analysis including age in step 1; results remained similar.

## Discussion

The present study investigated the phonological and orthographic spelling development of children with dyslexia by analyzing phonological, morphological, and orthographical spelling errors both before and after a phonics through spelling intervention. The predictive role of semantics for children with dyslexia was studied as an addition to the phonological core deficit.

In line with the first hypothesis, children with dyslexia made more phonological, morphological, and orthographic errors compared to typically developing children. These results confirm previous research (Bourassa & Treiman, 2003; Bourassa et al., 2006; Cassar et al., 2005).

In accordance with our second hypothesis, we found that orthographic spelling is more difficult for all spellers. This finding confirmed that more specific knowledge of rules and orthographic patterns is necessary for orthographic spelling (see Allen, 2019). Both children with dyslexia and typically developing children made proportionally most morphological errors, followed by orthographic and phonological errors. In addition, when comparing the relative differences between children with dyslexia and typically developing children, results showed that children with dyslexia differed most from typically developing children on phonological errors compared to morphological and orthographic errors. The fact that children with dyslexia struggle more than typically developing children with phonological errors follows from their phonological deficit which prevents children with dyslexia from building strong bidirectional connections between phonological and orthographic representations (Cassar et al., 2005; Snowling, 1998).

In compliance with our third hypothesis, we found that for children with dyslexia, phonological, morphological, and orthographic errors were negatively associated with their level of semantics. The importance of semantics for both phonological and orthographic spelling is in line with the lexical quality hypothesis (Perfetti, 2007); semantics could be seen as a compensatory factor for both phonological and orthographic spelling. We showed that semantics is not only important for reading (Nation & Snowling, 2004) but for phonological and orthographic spelling as well. This is in line with the connectionist model of Plaut et al. (1996) in which literacy development was described in terms of a division of labor between interacting phonological and semantic pathways. Variation in both phonological and semantic processing could be related to individual differences in literacy development, whereas children with dyslexia rely on contributions from the semantic pathway because of their poorly developed phonological pathway (Nation & Snowling, 1998; Snowling, 2000). However, longitudinal studies are necessary to understand the complex interplay between phonological and semantic abilities as been indicated by Laing and Hulme (1999).

With regard to our fourth hypothesis, we found a decline in the amount of errors in all three error categories after a phonics through spelling intervention. The decline is quite alike for all three categories and the relative differences between the three categories stayed the same: children with dyslexia still made relatively most morphological errors, followed by orthographic errors and lastly phonological errors (based on percentages of the total amount of possible errors in that category). These results are all in line with the spelling improvements that were found after interventions including phonics, morphological, or orthographic instruction (Galuschka et al., 2020) and illustrate that phonics through spelling interventions have a positive effect on both phonological and orthographic spelling development.

Consistent with our fifth hypothesis, we found that for children with dyslexia, the decline in phonological, morphological, and orthographic errors during the phonics through spelling intervention were associated with their level of semantics. Semantics influence the spelling development over time and together with the above-mentioned findings, this leads to the conclusion that children with better semantics do not only perform better in spelling in general but gain more during a phonics through spelling intervention as well.

The present study can be seen as a first step in uncovering the development of phonological and orthographic spelling and the role of semantics in this spelling development of children with dyslexia. Some limitations should be acknowledged at this point along with directions of future research. First, results should be interpreted with caution as children could make more phonological errors compared to morphological and orthographic errors. Although we used a standardized Dutch dictation task, it is recommended for future research to consider dictations tasks in which errors will be more evenly distributed among these three categories and in which higher levels are included as well. This way, the control group will show more normally distributed scores without floor effects. Future research could find out whether the predictive role of semantics for spelling errors is specific for children with dyslexia or not. Second, we only followed the children with dyslexia over time and did not incorporate a (randomized) control group to evaluate the effects of the intervention. In future research, this latter group could also be followed over time in order to find out whether the effect of semantics on the spelling development over time is typically for children with dyslexia. Follow-up studies could include both skill-based and word-based effects of semantics in order to find out which benefits children most. Third, future research could consider to include the actual time spent on each spelling error category during the intervention in order to see how this influenced the spelling error decline. Fourth, although this study checked for the amount and frequency of the homework that comes with the intervention, there could be an effect of home environment on the change due to intervention, for instance, due to differences in socioeconomic status. Follow-up studies focusing on the influence of the home environment are recommended.

It can be concluded that in general, orthographic spelling is more difficult than phonological spelling, but that children with dyslexia differ far more from typically developing children in the proportion of phonological errors compared to the difference in the proportion of morphological and orthographic errors. This is in line with their phonological deficit and implies that children with dyslexia need an intervention that stimulates both phonological and orthographic spelling development, but especially phonological errors since the differences with typically developing children are biggest. This study contributes to the existing literature by demonstrating that children with dyslexia with strong semantic representations appear to make less phonological, morphological, and orthographic spelling errors, compared to children with dyslexia with less developed semantic representations. Our study shows that a phonics through spelling intervention benefits both phonological and orthographic spelling development quite evenly among children with dyslexia but that there is a positive additional effect of semantics on the progress during the intervention. Children with better semantics make less errors to begin with but make more progress during the intervention as well. Promising effects of integrating semantics in spelling interventions were found before (Ouellette, 2010; Ouellette & Fraser, 2009). Therefore, it is recommended to stimulate the semantic development of children that are at risk for dyslexia at an early stage and throughout their school carrier in order to help them build strong semantic networks to compensate in their spelling development. Furthermore, semantics could be stimulated during interventions as well in order to stimulate spelling development even further.

## Data Availability

There is a dataset associated with this submission which can be requested from the first author.

## Appendix

**Table 6.** Classification of errors in phonological, morphological, and orthographic errors and corresponding error descriptions

Type of error	Explanation, examples, and total error calculation
Phonological errors	
Phoneme addition	The addition of one or more grapheme per place (e.g., zons [sun] instead of zon).
Phoneme deletion	The deletion of a grapheme (e.g., zo [so] instead of zon [sun]).
Long vowel in closed syllable swap	A long vowel in a closed syllable is written as a short vowel (e.g., ram [ram] instead of raam [window]) or as another long vowel (e.g., room [cream] instead of “raam” [window]).
Long vowel in open syllable swap	A long vowel in an open syllable is swapped for the wrong vowel (e.g., ro-men [skimming] instead of ramen [windows]).
Short vowel swap	A short vowel is swapped for the wrong vowel (e.g., rum [rum] instead of ram [ram]).
Two-character vowels swap	A two-character vowel (e.g., ie) is swapped for the wrong two-character vowel (e.g., “puis” [no Dutch meaning] instead of poes [cat]), or written incorrectly (e.g., “peos” [no Dutch meaning] instead of poes [cat]).
Consonant swap	A consonant is swapped for the wrong consonant (e.g., poek [no Dutch meaning] instead of “poes” [cat]).
Morphological errors	
End d/t	When words end with a /t/, it can be written with “t” or “d” on the end of the word (e.g., hond [dog], boot [ship]). By making a word plural, it is possible to hear a “t” or a “d” (e.g., hon*d*en, bo*t*en). Whatever consonant is heard in the plural form must be written in the singular word as well. Errors: write a “t” instead of a “d” or vice versa (e.g., hont instead of hond).
Open syllables long vowel	When a syllable ends with a long vowel, this is written short (raa-men ➔ ra-men [windows]). Errors: write a long vowel (e.g., boomen instead of bomen).
Open syllables short vowel	When a syllable ends with a short vowel, the next consonant is geminated (ki-pen ➔ kippen [chickens]). Errors: forget to geminate the following consonant (e.g., kipen instead of kippen).
gt/cht	Words with /gt/, which can be written as “gt” or “cht” (e.g., zaagt [sweeps], lucht [air]). Whenever a short sound vowel is placed before /gt/, then a “cht” needs to be written (l*u*cht). There are three exceptions: ligt (lays down), legt (put), zegt (says). Errors: write a “gt” instead of “cht” (e.g., vrugt instead of vrucht) or vice versa (veecht instead of veegt) or make mistakes in exception words. (e.g., zecht instead of zegt).
Orthographical errors	
aai/ooi/oei	The letter clusters aai/ooi/oei are pronounced as aaj/ooj/oej. Errors: write the j instead of the i.
eeuw/ieuw/uw	The letter clusters eeuw/ieuw/uw are pronounced as iw/iew/uuw, for instance in leeuw (lion), nieuw (new), and duw (push). Errors: writings like “leew” instead of “leeuw.”
i/ie/y	The sound /ie/ can be written in three ways. The two-character vowel “ie” and “y” are always pronounced like “ie,” for instance in “gieter” (watering can) and “systeem” (system). The letter “i” is pronounced like “ie” or “i” depending on the word, for instance “liter” (/lietur/) and dieper (/diepur/, deeper). It needs to be learned by heart whether to write the “ie,” “i,” or “y” in case of an /ie/. Errors: writings like “lieter”/“lyter” instead of “liter.”
g/ch	The sound /g/ (not followed by a “t” ➔ see gt/cht rule) can be written in two ways: “g” or “ch.” It needs to be learned by heart whether to write the “g”/“ch.” Errors: writings like “zach” (no Dutch meaning) instead of “zag” (saw).
c/k/s	The sound /k/ can be written as “k” or “c” and the sound /s/ can be written as “s” or “c.” It needs to be learned by heart whether to write the “k”/“c” and “s”/“c.” Errors: writings like “lieter”/“lyter” instead of “liter.”
f/v and s/z	Although there is a difference in sounds of the “f” and “v” and “s” and “z” sound alike, for instance in fiets (bike) and vlag (flag). For many children, this is therefore an orthographic category. Errors: writings like “viets” instead of “fiets” or “see” instead of “zee.”
ou/au/ouw/auw and ei/ij	The sound /au/ can be written in four ways: “au”/“ou”/“auw”/“ouw,” and /ij/ as “ij” or “ei.” For instance in “saus” (sauce), “kous” (stocking), “rauw” (raw) or “mouw” (sleeve). It needs to be learned by heart which letter combination to write. Errors: writings like “sous” instead of “saus” or “trijn” instead of “trein.”
Schwa	A reduced vowel in unstressed syllables that in Dutch is pronounced like /u/. It may be written using any of the following letters: “e,” as in mur*e*n (walls) “i,” as in grapp*i*g (funny) “ij,” as in moeil*ij*k (difficult) The schwa-parts of words need to be learned by heart. Errors: writings like “grappug”/“grappeg” instead of “grappig.”

**Table 7.** Removed types of errors

Type of error	Explanation, examples, and total error calculation
Tone discoloration	The letters “r” and “l” can discolorate the tone of certain grapheme or grapheme-clusters such as ee, oo, eu, ij, and ei. For instance, “veel” (a lot of) sounds like /vil/ with a slightly long stretched short vowel /i/. Errors: mostly the tone discoloration is not recognized and the short vowels are written down. For instance, “vil” instead of “veel.” Deleted because it could be classified as both morphological as orthographic.
Adhesive letters	In certain parts in the Netherlands, a schwa is added in between two consonants in the letter clusters with “r” (-rg/-rp/-rm/-rk/-rf) and with “l” (-lg/-lp/-lm/-lk/-lf). For instance, the word “tulp” (tulip) is pronounced /tulup/. A schwa is added between two consonants. Errors: the schwa is sometimes added in the written word, for instance “tulup” instead of “tulp.” Deleted because it could be classified as both morphological as orthographic or phonological error.
-ng/-nk	The grapheme-combination “-ng” and “-nk” are difficult because the sound is different from the known grapheme’s “n,” “g,” and “k.” The “ng” forms a new sound and the “nk” is pronounced like “ngk.” The sound and grapheme-combination have to be known by heart. Errors: mostly the “ng” is written like “n,” for instance “krin” instead of “kring” (circle). The “nk” is mostly written like “ngk,” for instance in “flingk” instead of “flink” (brave). Deleted because some schools treat it as a grapheme-phoneme correspondence whereas others see it as an orthographic word to remember.
