# Correction to: Semantics impacts response to phonics through spelling intervention in children with dyslexia

**DOI:** 10.1007/s11881-022-00263-3

**Published:** 2022-07-16

**Authors:** R. van Rijthoven, T. Kleemans, E. Segers, L. Verhoeven

**Affiliations:** 1 Behavioural Science Institute, Radboud University, Nijmegen, The Netherlands; 2 OPM, Nijmegen, The Netherlands

**Correction to** **:** **Annals of Dyslexia (2021) 71:527–546**

**https://doi.org/10.1007/s11881-021-00233-1**

Clerical errors were found in the data presented in the original article. These were in the calculation of the raw and percentile scores of phonological, morphological and orthographic errors. The authors reran all analyses, and although the means changed, most results remained the same. In Tables 1, 2, 3, 4 and 5, the quotations and the appendix found hereafter, the relevant corrections are marked in bold and brief explanations are given in italics and enclosed in parentheses:

**Table 1:** Descriptive statistics for children with dyslexia at pretest (*N* = 52) and typically developing children (*N* = 105)

		Children with dyslexia		Typically developing children	
		*M*	SD	*M*	SD
Outcome measures					
Word spelling pretest	Raw scores	43.15	23.54	80.23	20.95
Phonological errors	Raw scores	**103.21**	**160.67**	1.98	2.50
	Percentages	**16.28**	**25.22**	0.32	0.41
Morphological errors	Raw scores	**6.77**	**4.37**	1.88	2.14
	Percentages	**40.47**	**25.64**	11.72	13.36
Orthographical errors	Raw scores	**17.44**	**22.17**	1.00	1.61
	Percentages	**20.96**	**26.18**	1.27	2.04
Predictor measures					
Phonological awareness					
Spoonerism	Raw scores	2.80	3.35	6.99	3.40
Phoneme deletion	Raw scores	7.96	2.35	9.99	1.74
Rapid automatized naming					
Letter naming	Raw scores	41.63	10.944	31.00	10.37
Digit naming	Raw scores	37.79	11.26	28.21	6.98
Verbal working memory	Raw scores	3.92	1.31	4.59	1.65
Semantics					
Information	Raw scores	12.58	3.27	12.64	3.23
Similarities	Raw scores	14.27	2.42	12.49	3.68
Vocabulary	Raw scores	30.23	6.14	28.22	6.94
Comprehension	Raw scores	20.60	5.52	16.60	5.62

**Table 2:** Pearson correlations between the outcome measures (i.e., word spelling, phonological errors, morphological errors, orthographical errors) and predictor measures (i.e., phonological awareness, rapid automatized naming, verbal working memory and semantics). Below the diagonal: children with dyslexia (*n* = 52); above the diagonal: typically developing children (*n* = 105)

	1.	2.	3.	4.	5.	6.	7.	8.	9.	10.	11.	12.
1. Word spelling raw scores pretest	-	− .585**	− .747**	− .617**	.610**	− .418**	.426**	.563**	-	-	-	-
2. Percentages phonological errors	− .768**	-	.392**	.370**	− .491**	.360**	− .146	− .343**	-	-	-	-
3. Percentages morphological errors	− .907**	.839**	-	.564**	− .519**	.241*	− .227*	− .451**	-	-	-	-
4. Percentages orthographical errors	− .810**	.992**	.857**	-	− .337**	.323**	− .242*	− .283**	-	-	-	-
5. Phonological awareness	.305*	− .245	− .293*	− .2**63**	-	− .255**	.317**	.528**	-	-	-	-
6. Rapid automatized naming	− .378**	.239	.3**90**	.25**6**	− .212	-	− .231*	− .318**	-	-	-	-
7. Verbal working memory	.294*	− .2**30**	− .2**47**	− .2**35**	.088	.040	-	.410**	-	-	-	-
8. Semantics	.652**	− .456**	− .665**	− .491**	.345*	− .359**	.085	-	-	-	-	-
9. Change per session phonological errors	.720**	**−** .893**	− .808**	− .878**	.315*	− .250	.275	.526**	-	-	-	-
10. Change per session morphological errors	.627**	− .598**	− .776**	− .580**	**.093**	− **.185**	.2**01**	.421**	.731**	-	-	-
11. Change per session orthographical errors	.740**	− .879**	− .812**	− .875**	.325*	− .2**90**	.2**48**	.537**	.990**	.713**	-	-
12. Change per session spelling percentiles	− .291*	− .2**11**	− .2**8**7	− .2**43**	.305*	− .305*	.02**7**	.363**	.292*	**.236**	.303*	-

*p < .05; **p < .01; ***p < .001

**Table 3:** Cognitive predictors of spelling errors in children with dyslexia (*N* = 52)

		Phonological errors		Morphological errors			Orthographical errors	
		*ΔR*^*2*^	β		*ΔR*^*2*^	β	*ΔR*^*2*^	β
Step 1		.151			.268**		.168*	
	Phonological awareness		− 2.71**1**			− **2.965**		− **3.045**
	Rapid automatized naming		2.7**38**			4.751**		3.03**1**
	Verbal working memory		− 7.0**83**			− 7.986*		− 7.**467**
Step 2		.123**			.275***		.143**	
	Phonological awareness		− 1.0**71**			− 0.**478**		− 1.**208**
	Rapid automatized naming		1.19**4**			**2.411**		1.3**02**
	Verbal working memory		− 6.3**12**			− 6.816*		− 6.6**02**
	Semantics		− 2.803**			− 4.250***		− 3.140**
Total *R*^2^_adj_		.211**			.503***		.250**	

*p < .05; **p < .01; ***p < .001

**Table 4:** Descriptive statistics from both pre- and posttest on all dependent measures, the results on the *t*-tests for paired samples, Cohen’s *d*, and the change per session

	Pretest (*n* = 52)		Posttest (*n* = 46)		Difference scores (*t*-tests)		Change per session	
	*M*	SD	*M*	SD	*t*	*d*	*M*	SD
Word spelling percentiles	5.40	10.50	13.25	15.10	3.41***	0.60	.26	0.5**7**
Percentage of phonological errors	16.**27**	25.2**2**	1.9**6**	11.07	− 4.34***	0.73	− .62	0.98
Percentage of morphological errors	**40.47**	2**5.64**	1**3.61**	1**6.34**	− 8.10***	**1.25**	**− 1.05**	**0.90**
Percentage of orthographical errors	20.**96**	26.**18**	3.**85**	12.8**5**	− 4.95***	0.**82**	− .72	**0.99**

*p < .05; **p < .01; ***p < .001

**Table 5:** Cognitive predictors of the change in spelling errors during the interventions (*N* = 45)

		Change in phonological errors		Change in morphological errors		Change in orthographical errors	
		*ΔR*^*2*^	β	*ΔR*^*2*^	β	*ΔR*^*2*^	β
Step 1		.204*		.0**86**		.213*	
	Phonological awareness		0.**142**		0.0**17**		0.**146**
	Rapid automatized naming		− 0.**109**		− 0.0**91**		− 0.**131**
	Verbal working memory		0.**338**		0.2**54**		0.3**05**
Step 2		.152**		.149**		.154**	
	Phonological awareness		0.0**59**		− 0.0**58**		0.0**62**
	Rapid automatized naming		− 0.0**55**		− 0.0**42**		− 0.0**76**
	Verbal working memory		0.**266**		0.18**9**		0.**232**
	Semantics		0.124**		0.113**		0.126**
Total *R*^2^_adj_		.292**		.235**		.367**	

*p < .05; **p < .01; ***p < .001

…The mean length of the intervention was 27.0**4** weeks (SD = 4.**93**)….

…made higher percentages of errors in all three categories (phonological errors *t*(**51**) = **4.562**, *p* < .001, *d* = **0.89**; morphological errors *t*(**65**) = **7.589**, *p* < .001, *d* = **1.41**; orthographic errors *t*(51) = 5.**416**, *p* < .001, *d* = 1.**05**). Regarding the predictive measures children with dyslexia scored weaker compared to typically developing children on phonological awareness measures (spoonerism ***t*(154) = − 7.249, *p* < .001, *d* = 0.98**; phoneme deletion ***t*(78) = − 5.483, *p* < .001, *d* = 1.24**).

…the main effects of Errortype, χ^2^ (2) = **368.098**, *p <* .001. Therefore, degrees of freedom were corrected using Greenhouse–Geisser estimates of sphericity (*ε* = .53 for Errortype). There was a main effect of Errortype, *F*(1.0**48**, 162.**440**) = **213.914**, *p* < .001, η^2^_p_ = **.580**, as well as an interaction between Errortype and Group, *F*(1, 155) = **25.245**, *p* = <**.001**, η^2^_p_ = **.140**. Children with dyslexia made more phonological (*t* (**51**) = **4.562**, *p* < .001, *d* = 0.**89**), morphological (*t*(**65**) = **7.589**, *p* < .001, *d* = **1.41**) and orthographic errors (*t* (**51**) = 5.**416**, *p* < .001, *d* = 1.**05**) than typically developing children. The interaction suggests that this difference tended to be more pronounced in the phonological errors. Indeed, planned contrasts revealed that the difference between the two groups is significant for phonological and orthographic errors (*F*(1,155) = **78.42**, *p* < .001), phonological and morphological errors (*F*(1,155) = 7**8.415**, *p* < **.001**) and morphological and orthographic errors (*F*(1,155) = **17.22**, *p* = **<.001**.

…The results of the analyses (see Table 3) showed that rapid naming **and verbal working memory** predicted morphological errors (step 2). In step 2, the effect of rapid naming on morphological errors was no longer present. Higher scores on semantics resulted in less phonological, morphological and orthographical errors, and **higher scores on verbal working memory resulted in less morphological errors**.

To check whether age had any impact on the results, we reran the hierarchical regression analysis including age in Step 1 and the predictor measures in steps 2 and 3; all results remained similar.

…Regarding the relative differences in change per session, results showed no differences between phonological and morphological (*t*(45) = **4.159**, *p* = **<.001**, *d* = 0.46) and morphological and orthographic errors(*t*(45) = **− 3.098**, ***p* = .003**, *d* = 0.35) and phonological and orthographic errors ((*t*(45) = 4.**613**, *p* < .001, *d* = 0.10). The change per session is **highest** for orthographical errors, **followed by orthographical** and phonological errors….

…In compliance with our third hypothesis, we found that for children with dyslexia **morphological errors were negatively associated with verbal working memory** and phonological, morphological and orthographic errors were negatively associated with their level of semantics. **The impact of verbal working memory on morphological errors is also highlighted in the Morphological Pathways Framework, suggesting that additional working memory is needed in these cases (Levesque et al., 2021)**. The importance of semantics for both phonological and orthographic spelling is in line with the lexical quality hypothesis (Perfetti, 2007); semantics could be seen as a compensatory factor for both phonological and orthographic spelling….

…This study contributes to the existing literature by demonstrating that children with dyslexia with strong semantic representations, appear to make less phonological, morphological and orthographic spelling errors, compared to children with dyslexia with less developed semantic representations **and children with dyslexia with better developed verbal working memory make less morphological errors compared to children with dyslexia with better developed verbal working memory**. Our study shows that a phonics through spelling intervention benefits both phonological and orthographic spelling development quite evenly among children with dyslexia but that there is a positive additional effect of semantics on the progress during the intervention….

Reference added:

Levesque, K. C., Breadmore, H. L., & Deacon, S. H. (2021). How morphology impacts reading and spelling: Advancing the role of morphology in models of literacy development. *Journal of Research in Reading*, *44*(1), 10-26. http://dx.doi.org/10.1111/1467-9817.12313

In addition we found four mistakes in Appendix A.

**Table:** 

End d/t	When words end with a /t/ it can be written with ‘t’ or ‘d’ on the end of the word (e.g., hond [dog], boot [ship]). By making a word plural it is possible to hear a ‘t’ or a ‘d’ (e.g., hon*d*en, bo*t*en). Whatever consonant is heard in the plural form must be written in the singular word as well. Errors: write a ‘t’ instead of a ‘d’ or vice versa (e.g., hont instead of hond).
Open syllables long vowel	When a syllable ends with a long vowel this is written short (raa-men → ra-men [windows]) Errors: write a long vowel (e.g., boomen **[trees]** instead of bomen).
gt/cht	Words with /gt/, which can be written as ‘gt’ or ‘cht’ (e.g., zaagt [sweeps], lucht [air]). Whenever a short sound vowel is placed before /gt/, then a ‘cht’ needs to be written (l*u*cht). There are three exceptions: ligt (lays down), legt (put), zegt (says). Errors: write a ‘gt’ instead of ‘cht’ (e.g., vrugt instead of vrucht) or viceversa (veecht **[wipes]** instead of veegt) or make mistakes in exception words. (e.g., zecht instead of zegt).
Open syllables short vowel	(*This category needs to be removed. It was not part of block 5-20.*)
c/k/s	The sound /k/ can be written as ‘k’ or ‘c’ and the sound /s/ can be written as ‘s’ or ‘c’. It needs to be learned by heart whether to write the ‘k’/’c’ and ‘s’/’c’. **Errors: writings like ‘creeft’ (lobster) instead of ‘kreeft’.**

